# 经气管镜电圈套器联合*CO_2_*冷冻及氩等离子体凝固等治疗77例气道内肿瘤和息肉

**DOI:** 10.3779/j.issn.1009-3419.2013.06.04

**Published:** 2013-06-20

**Authors:** 洪武 王, 冬妹 李, 楠 张, 珩 邹, 洁莉 张, 晶 李, 素娟 梁

**Affiliations:** 100028 北京，煤炭总医院肿瘤内科 Department of Medical Oncology, Beijing Coal General Hospital, Beijing 100028, China

**Keywords:** 气道内肿瘤, 息肉, 电圈套器, CO_2_冷冻, 氩等离子体凝固, Airway neoplasms, Granulomas, Electrocautery snare, Cryosurgery, Argon plasma coagulation

## Abstract

**背景与目的:**

采用电圈套器联合CO_2_冷冻等治疗气道内大的肿瘤或息肉，探讨其疗效和安全性。

**方法:**

77例大气道内肿瘤或息肉患者（恶性肿瘤70例，良性病变7例）在硬质镜引导下，结合电子支气管镜，利用电圈套器联合CO_2_冷冻等进行治疗。

**结果:**

77例患者进行电圈套器治疗85例次。病变位于右侧支气管42.9%，主气管38.3%和左侧支气管21.4%，少数位于亚段支气管开口。恶性肿瘤混合型占89.7%，单纯管内型仅占10.3%。圈套器治疗前气道内堵塞约4/5，术后约1/5；KPS评分和气促指数治疗后均有明显改善。未发生大出血、穿孔等并发症。

**结论:**

电圈套器联合CO_2_冷冻等治疗气道内肿瘤或息肉，安全、有效、快速。

## Abstract (en)

**Background and objective:**

To observe the clinical effects and safety of endobronchial electrocautery snare combined with CO_2_ cryosurgery for the treatment of tracheobronchial obstructive lesions.

**Materials and methods:**

Seventy-seven patients with airway tumor or granuloma were retrospectively reviewed, including 70 malignant airway obstruction and 7 benign airway lesions, for the treatment of endobronchial electrocautery snare plus CO_2_ cryosurgery and argon plasma coagulation (APC).

**Results:**

Eighty-five endobronchial snares were performed in 77 cases. 42.9% of the obstructive lesions were located in right bronchial orifice, 38.3% in main trachea 21.4% in left bronchial orifice. 89.7% of the malignant tumor was mixed type of lesions (endobronchial plus bronchial or external bronchial), only 10.3% was endobronchial. Obstructive stenosis was significant relieved after snare (80% before *vs* 20% after, *P* < 0.01) in all patients. Karnofsky Performance Status (KPS) and shortbreath score were obviously improved after snare. There was no severe complications related to the procedures.

**Conclusion:**

Endobronchial electrocautery snare is an effective and safe approach for tracheobronchial obstructions with few complications.

近年来随着电子支气管镜下介入治疗技术的发展，大气道内肿瘤的消除并不困难，常用的方法有高频电刀、氩等离子体凝固（argon plasma coagulation, APC）、激光、CO_2_冷冻、内支架置入等。电圈套器是一种特殊类型的高频电刀，广泛应用于消化道肿瘤或息肉的电切摘除^[1]^，在气道息肉或肿瘤的治疗中也有应用^[2, 3]^，但远未普及。作者总结77例临床应用电圈套器联合CO_2_冷冻等治疗气道内大肿瘤的经验，供同行参考。

## 1 材料与方法

### 1.1 临床资料

回顾性分析自2010年2月-2012年3月收治的77例大气道内肿瘤或息肉患者，年龄33岁-84岁（平均年龄60.9岁±1.3岁），其中男性54例，女性23例。

气道内病变性质：气道内恶性肿瘤70例，其中原发癌30例，包括鳞癌11例，腺样囊性癌8例，腺癌、腺鳞混合癌、小细胞癌（small cell lung cancer, SCLC）和类癌各2例，鳞癌合并SCLC、肉瘤样癌、粘液表皮样癌各1例。气管转移癌40例，来源于肺癌29例（其中鳞癌24例，腺癌3例，腺鳞混合癌和SCLC各1例，食管癌5例，甲状腺癌3例，肾癌2例，大肠癌1例）。

气道内良性病变7例，其中4例良性肿瘤（血管球瘤2例，脂肪瘤和纤维瘤各1例），气管切开后气道息肉2例、气道淀粉样变1例。

所有患者均经胸外科大夫会诊，认为不适合手术切除。

本方案经医院伦理委员会同意，并经患者本人和/或家属签署知情同意书，愿意接受气管镜介入治疗。

### 1.2 操作方法^[4]^

#### 1.2.1 气管镜及配套设备

##### 1.2.1.1 电子支气管镜（简称软镜）

所用软镜为日本PENTAX-EPM3500。按电子支气管镜操作常规进行，术前给予无痛镇静及局部喷射麻醉，术中持续静脉镇静麻醉。

##### 1.2.1.2 硬质镜

所用硬质镜为德国Karl Storz（Tutlingen）。操作在手术室进行。麻醉前面罩吸氧，预氧合5 min-10 min。术前10 min静脉滴注阿托品0.5 mg或东莨菪碱0.3 mg，以抑制气道内过多的分泌物。术中需监测血氧饱和度、心电图、血压及呼吸运动等。患者诱导前5 min应用咪哒唑仑2 mg静注，随后静注芬太尼1 μg/kg^-2^ μg/kg，1%异丙酚（1 mg/kg^-2^ mg/kg）。然后给予肌松剂阿曲库铵0.5 mg•kg^-1^，待肌颤消失、下颌肌肉松弛后即可经口插入硬质镜。维持药物浓度为1%异丙酚1 mg/kg•h^-1^-2 mg/kg•h^-1^，瑞芬太尼0.1 μg/kg•min^-1^-0.2 μg/kg•min^-1^。然后接麻醉呼吸机及高频喷射通气，通过硬质镜后端的操作孔进行各种操作。

#### 1.2.2 气管镜介入治疗设备

氩等离子体凝固（APC）所用设备为德国产CESEL 3000型。将APC探针通过电子支气管镜活检孔伸出气管镜插入端（能见到探针标志为准），在距病灶0.5 cm以内时开始烧灼。APC输出功率为30 W-50 W，氩气流量为0.8 L/min-1.6 L/min。烧灼过程中勿需停止吸氧，但以间断烧灼为宜（每次5 s-10 s左右），时间不能太长，并不断用活检钳取出碳化凝固的组织（碳化的组织易燃烧着火）。

高频电发生器（PSD-20、UES-30）为日本奥林巴斯公司生产及电圈套器型为南京微创公司生产。电凝功率30 W-40 W。电切时将圈套器环绕肿瘤基底部，手拉紧收缩圈套器，然后启动高频电凝，通电时间5 s-10 s，即可切除肿瘤。再用光学活检钳或冷冻将切下的肿瘤取出。对基底部较大或不能圈套的肿瘤，则用冻切的方法。

冷冻机采用北京库兰医疗设备有限公司生产的冷冻治疗仪K300型和德国ERBE。软式可弯曲冷冻探头直径1.9 mm-2.3 mm，探针末端长度5 mm。冷源为液态二氧化碳。将冰冻探头的金属头部放在肿瘤表面或推进到肿瘤内，冷冻5 s-10 s，使其周围产生最大体积的冰球，在冷冻状态下将探头及其粘附的肿瘤组织取出，必要时再插入探头，直至将腔内的肿瘤全部取出。冻取后如有出血，则结合APC止血。

#### 1.2.3 电圈套器适应证

主要用于有蒂肿瘤或息肉的切除。也可用于宽基底的腔内肿瘤。特殊情况亦可用作电切刀。

#### 1.2.4 疗效判断

根据作者的经验，将中央型气道分为8个区^[5]^：主气管等分为3部分，自上而下为1、2、3区；隆突为4区；右主支气管为5区；右中间段支气管为6区；左主支气管近1/2段为7区，远1/2段为8区。不同的区域应采取不同的治疗方法。

气道狭窄再通的疗效判断标准^[6]^：完全有效（complete response, CR）：腔内病灶完全清除，功能恢复正常；部分有效（partial response, PR）：超过50%的狭窄管腔重新开放，功能检查大致正常，患者主观症状改善；轻度有效（mild response, MR）：狭窄改善不足50%，但经引流，狭窄远端肺部炎症消散；无效（no response, NR）：病变未消除，狭窄未缓解。

气促分级采用美国胸科协会气促评级标准^[7]^：0级：正常；1级：快步走时出现气促；2级：平常速度步行时出现气促；3级：平常速度步行时因出现气促而停止步行；4级：轻微活动后出现气促。

### 1.3 统计处理

采用SPSS 11.0统计软件包进行分析，采用*t*检验分析组间差异。生存时间起点以接受电圈套器的第1 d开始计算，术后3个月随访1次，随访至少半年。生存率用*Kaplan-Meier*公式计算。*P* < 0.05为差异有统计学意义。

## 2 结果

### 2.1 圈套的病变所处气道的部位

由表 1可见，70例恶性肿瘤患者进行了78例次大气道电圈套器等治疗，肿瘤部位发生于右侧支气管（5区+6区）30个（42.9%），主气管（1区+2区+3区）24个（38.3%），左侧支气管（7区+8区）15个（21.4%）。原发和继发恶性肿瘤两组间无明显差异，均以3区和5区最常见。7例良性病变发生部位无规律可言。

**1:** 圈套的病变所处气道的部位 Airway sites treated by electric snare

Airway sites	32 sites with primary airway tumor (*n1*/%)	46 sites with secondary airway tumor (*n2*/%)	7 sites with beingn airway diaeases (*n3*/%)
1	2 (5.4)	1 (2.2)	1 (14.3)
2	3 (8.1)	2 (4.3)	1 (14.3)
3	5 (13.5)	11 (23.9)	1 (14.3)
4	0 (0)	1 (2.2)	1 (14.3)
5	7 (18.9)	14 (30.4)	1 (14.3)
6	5 (13.5)	4 (8.7)	0 (0)
7	2 (5.4)	4 (8.7)	1 (14.3)
8	5 (13.5)	4 (8.7)	0 (0)
Right upper bronchus	2 (5.4)	0 (0)	0 (0)
Left lower bronchus	1 (2.7)	0 (0)	0 (0)
Left upper bronchus	0 (0)	1 (2.2)	1 (14.3)

根据病变所处管壁上的位置，30例原发性恶性肿瘤有32个病变部位：管内+管壁+管外20个（62.5%），管内+管壁8个（25%），管内4个（12.5%）。40例转移性肿瘤有46个病变部位：管内+管壁+管外20个（43.5%），管内+管外22个（47.8%），管内4个（8.7%）。转移性肿瘤组（管内+管壁）明显多于原发肿瘤组（*P* < 0.05），其它两组无明显差异。恶性肿瘤混合型（两种以上病变）占89.7%，单纯管内型仅占10.3%。

### 2.2 气管镜下所采用的方法

本组77例患者采用气管镜下圈套治疗85例次，其中只有3例次单独在电子支气管镜下完成，其余82例次均采用硬质气管镜结合电子支气管镜完成。30例原发肿瘤圈套治疗32例次，40例转移性肿瘤完成圈套治疗46例次。所有70例恶性肿瘤患者接受气管镜治疗平均每例3.8±0.4次。

### 2.3 圈套器治疗前后患者气道阻塞及临床状况的改善情况

由表 2可见，除良性病变组KPS圈套器治疗前后无明显差别，其余各项治疗指标圈套器治疗前后均有明显变化（*P* < 0.01）。

**2:** 圈套器治疗前后患者气道阻塞及临床状况的改善情况 Changes of airway obstruction and clinical situations before and after treatment

	Malignant tumor (*n*=70)	Benign diseases (*n*=7)
Obstruction (%)		
Before treatment	84.0±2.2^*^	80.0±10.6^*^
After treatment	22.2±3.3	22.9±10.4
KPS (%)		
Before treatment	62.9±2.2^*^	70.0±6.9
After treatment	80.6±1.6	82.9±8.1
Breathlessness score		
Before treatment	2.8±0.1^*^	2.4±0.4^*^
After treatment	1.3±0.1	0.7±0.4
Comparison between before and after treatment (^*^P < 0.01).		

圈套器治疗前气道内堵塞多较严重（图 1A，图 2A），用电圈套器结合冷冻可将肿瘤或息肉取出（图 1B，图 2B），术后见创面平整，周边发白、凝固，无穿孔发生，出血较少、可控。有蒂的肿瘤可一次性切除，较大的或宽基底的肿瘤需多次圈套器套扎，或与冷冻、APC等结合应用。治疗后气道内病变大多消失，管腔通畅（图 1C，图 2C）。

**1:**
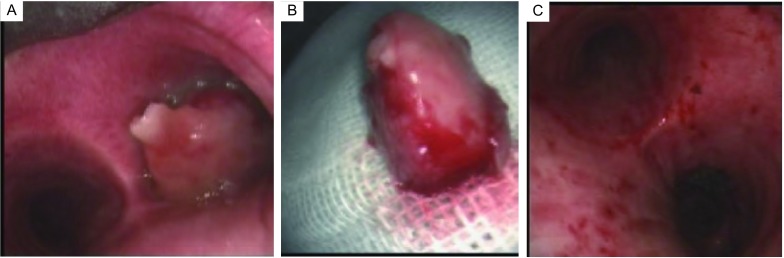
左下肺癌切除术后右主支气管内转移（男，60岁）。A：硬质气管镜下可见右主支气管开口被一肿物堵塞，表面光滑，质地柔软；B：用电圈套器结合CO_2_冷冻将右主支气管内肿物（1 cm×2 cm）取出；C：肿瘤取出后管腔通畅，其蒂部位于右主支气管内侧壁，残部用APC烧灼。 Right bronchus metastasis after left lower lobectomy with lung cancer (M, 60 yrs). A: A obstructive mass with smooth and soft could be found in right bronchus under rigid bronchoscopy; B: The mass (1 cm×2 cm) was removed by electric snare and CO_2_ cryoextraction; C: The right bronchus was reopened and the mass was originated from the inner wall of right bronchus.

**2:**
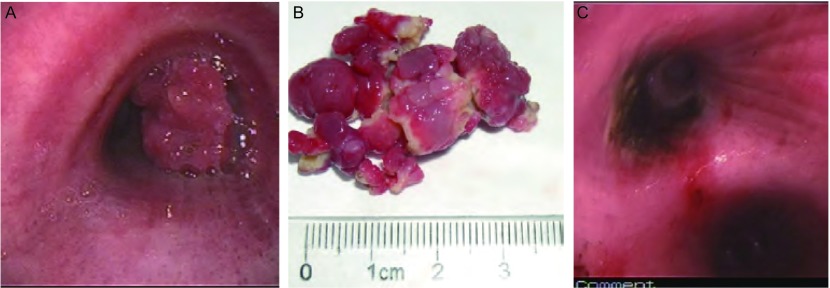
右主支气管炎性息肉（男，53岁）。A：气管镜下可见右主支气管开口被一菜花样肿物堵塞，表面呈结节状；B：先后3次用电圈套器结合CO_2_冷冻将右主支气管内肿物（2 cm×3.5 cm）取出，病理为炎性息肉；C：息肉取出后管腔通畅，其蒂位于右上支气管内侧壁，残部用APC烧灼。 Inflamatory granuloma in right bronchus (M, 53 yrs). A: A cauliflower-like and nodular mass was found in right bronchus under rigid bronchoscopy; B: A pathologically proved inflamatory granuloma was removed by 3 times of procedures with electric snare and CO_2_ cryoextraction; C: The right bronchus was reopened and the granuloma was originated from the inner wall of right bronchus. The remaining was destroyed by argon plasma coagulation (APC).

经治疗后，78例次恶性肿瘤CR 24例次（占30.8%），PR 47例次（60.3%），MR 7例次（9.0%）。有效率（CR+PR）为91%，临床获益率（CR+PR+MR）为100%。良性病变CR 4例（57.1%），PR 2例（28.6%），MR 1例（14.3%）。

根据*Kaplan-Meier*生存曲线，恶性肿瘤生存时间超过1年者占27.1%（原发气管癌与转移性气管癌相似）。中位生存时间为6个月，平均生存时间8.3个月（图 3）。

**3:**
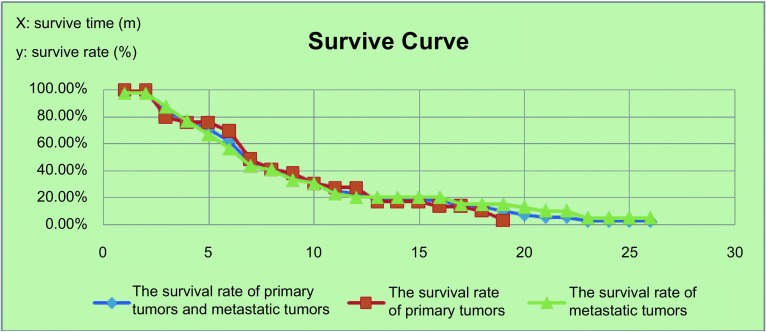
恶性气管癌患者生存曲线 Survive curve of patients with malignant airway tumor

## 3 讨论

高频电刀是一种将电能转换成热能，切除病变组织或消融的热凝切技术。我国于1984年开始采用经支气管镜高频电刀对气管支气管良恶性肿瘤、炎性肉芽肿等进行治疗^[8]^。电圈套器是一种特殊的高频电刀，主要用于有蒂肿瘤或息肉的切除。电圈套器是消化镜必不可少的工具，已成为一个重要的产业。但在呼吸领域，电圈套器只有零星的应用和报告，远未形成产业。

气道内大的肿瘤或息肉是呼吸内镜大夫最棘手的问题，由于瘤体较大，一般的活检钳难以抓取。既往作者对气道内大的肿瘤或息肉主要采用APC、冻取等方式治疗^[4, 9]^，但有出血及窒息等风险，且手术时间长，治疗次数多。

本文所套取的病变组织以右主支气管最常见，次为主气管下段。肿瘤或息肉均位于管腔内。单纯管腔内病变较少，大多为混合型（管内型合并管壁型或合并管外型）。

电圈套器最适合于有蒂的肿瘤或息肉。电圈套器主要由圈套钢丝、塑料套管和手柄组成，圈套器张开后的形状多呈椭圆形，也有六角形、新月形等。治疗时调整好镜身位置，从钳道伸出圈套器，根据病变的大小打开圈套，套住病变，以圈套器外套管的前端贴住病变，再逐渐收紧圈套，根据需要套住病变的大小。套住后可稍微前后移动圈套器，在通电中逐步收紧圈套器，直至病变切除。对有蒂肿瘤或息肉，将圈套器套于蒂上并通电后，即可将组织电凝切除，一般不会引起出血。对切下的较大的组织，可用三爪异物钳取出或冻取。对于无蒂息肉，电灼时应先以高渗盐水或1:10, 000肾上腺素溶液注入肿瘤或息肉基底部1点-2点，每点1.0 mL，然后圈套切除隆起的组织。对基底部较大或不能直接圈套的肿瘤，可将电圈套器电凝探头稍突出鞘管，置于病灶上，通电10 s-30 s，多次点击电凝，使病灶凝固、炭化（相当于电切刀的作用）。或将组织切割成多块，以便于圈套器套取。

作者既往用气管镜治疗气道内肿瘤，一般需5.9次^[10]^。本组资料只需3.8次，每次所需的时间也明显减少。经圈套器等治疗后，气道内阻塞明显减轻，临床症状明显改善，气促评分明显减低。经综合治疗后，恶性肿瘤CR达30.8%，PR达60.3%，MR达9.0%，有效率91%，临床获益率100%。良性病变CR达57.1%，PR达28.6%，MR达14.3%。

治疗过程均较安全，无1例发生大出血、穿孔或死亡等严重并发症。恶性肿瘤中位生存时间6个月，超过1年者占27.1%。

总之，高频电圈套器治疗气道阻塞，疗效明显，且费用较低，手术创伤小，并发症少，术后恢复快，值得临床广泛应用。

## References

[1] Wang P, Wu J, Huang XD (2010). Treatment of large colorectal polyps with wide peduncle by nylon endoloop ligature and/or clamping with titanium clips in combination with colonoscopy-assisted high-frequency electric snare:an analysis of 156 cases. Shijie Hua Ren Xiao Hua Za Zhi.

[2] Zhen YQ, Teng L, Wu LP (2008). Investigation of electric bronchoscope treatment for benign trachea tumor with high frequency knife. Zhongguo Nei Jing Za Zhi.

[3] Ishibashi H, Akamatsu H, Kikuchi M (2003). Resection of endobronchial hamartoma by bronchoplasty and transbronchial endoscopic surgery. Ann Thorac Surg.

[4] Wang HW, Zhang N, Li DM (2011). Clinical analysis of interventional bronchoscopy for the treatment of malignant obstructive atelectasis. Zhongguo Fei Ai Za Zhi.

[5] Wang HW (2012). Applicatiron of bronchoscopy for the treatment of central airway stenosis. Guoji Hu Xi Za Zhi.

[6] Sneyd JR (2003). Remifentanil manual versus target-controlled infusion. Anesth Analg.

[7] Stulbarg MS, Adams L (1994). Textbook of respiratory medicine. Philadelphia; Saundersm.

[8] Li YP, Chen CS, Ye M (2007). Endoscopic treatment of benign central airway stenosis. Zhongguo Nei Jing Za Zhi.

[9] Wang HW, Zhou YZ, Li DM (2011). Video-assisted rigid bronchoscopy for the treatment of central lung neoplasma. Zhonghua Jie He He Hu Xi Za Zhi.

